# Mitochondrial tRNA Mutation and Regulation of the Adiponectin Pathway in Maternally Inherited Hypertension in Chinese Han

**DOI:** 10.3389/fcell.2020.623450

**Published:** 2021-01-22

**Authors:** Jing Bai, Qiang Ma, Yunfeng Lan, Yating Chen, Shanshan Ma, Jiaxin Li, Chuanbin Liu, Zihao Fu, Xu Lu, Yun Huang, Yang Li

**Affiliations:** ^1^Medical School of Chinese People's Liberation Army (PLA), Beijing, China; ^2^Department of Cardiology, The Sixth Medical Center, Chinese People's Liberation Army (PLA) General Hospital, Beijing, China; ^3^Hainan LANBO Health Management Co. Ltd., Sanya, China; ^4^Department of Gerontology, Union Hospital, Tongji Medical College, Huazhong University of Science and Technology, Wuhan, China

**Keywords:** mitochondrial tRNA, mutation, maternally inherited hypertension, adiponectin, Chinese Han

## Abstract

Some essential hypertension (EH) patients show maternal inheritance, which is the mode of mitochondrial DNA inheritance. This study examines the mechanisms by which mitochondrial mutations cause EH characterized by maternal inheritance. The study enrolled 115 volunteers, who were divided into maternally inherited EH (group A, *n* = 17), non-maternally inherited EH (group B, *n* = 65), and normal control (group C, *n* = 33) groups. A mitochondrial tRNA (15910 C>T) gene mutation was significantly correlated with EH and may play an important role in the pathogenesis of maternally inherited EH. Examining two families carrying the mitochondrial tRNA 15910 C>T mutation, which disrupted base pairing and may affect the stability and function of mitochondrial tRNA^Thr^, we find that the overall incidence of EH was 59.3% in the maternal family members and 90% in males, significantly higher than in the general population in China (23.2%), and that the EH began at a younger age in those carrying mitochondrial tRNA 15910 C>T. To reveal the mechanism through which mitochondrial tRNA 15910 C>T causes maternally inherited EH, we cultured human peripheral blood mononuclear cells from family A2 *in vitro*. We find that cells carrying mitochondrial tRNA 15910 C>T were more viable and proliferative, and the increased ATP production resulted in raised intracellular reactive oxygen species (ROS). Moreover, the mitochondrial dysfunction resulted in reduced APN levels, causing hypoadiponectinemia, which promoted cell proliferation, and produced more ROS. This vicious cycle promoted the occurrence of EH with maternally inherited mitochondrial tRNA 15910 C>T. The mitochondrial tRNA 15910 C>T mutation may induce hypertension by changing the APN, AdipoR1, PGC-1α, and ERRα signaling pathways to elevate blood pressure. We discover a new mitochondrial mutation (tRNA 15910 C>T) related to EH, reveal part of the mechanism by which mitochondrial mutations lead to the occurrence and development of maternally inherited EH, and discuss the role of APN in it.

## Introduction

Essential hypertension (EH) is the most common cardiovascular disease. The global prevalence of EH is increasing annually, and the latest national survey showed that the prevalence of hypertension in Chinese adults was 23.2% (Wang et al., 2018). EH is a complex disease caused by genetic susceptibility and environmental factors. Previous studies on EH genetics mainly focused on nuclear genes, which cannot fully explain the pathogenesis of EH (Levy et al., 2009; Padmanabhan et al., 2010). In the past decade, some patients with matrilineal EH have been found; matrilineal inheritance is unique to mitochondrial DNA (mtDNA) transmission. Wang et al. explored a large Chinese family, including 106 subjects, and found a significant correlation between mitochondrial tRNA^Ile^ 4263A>G mutation and EH (Wang et al., 2011). Zhou et al. report a mitochondrial tRNA^Met^ 4435A>G mutation that they believe affected the structure of mitochondrial tRNA and mitochondrial function, thereby inducing maternally inherited hypertension (Zhou et al., 2018). Previously, we found that the frequency and density of mtDNA variation in Chinese Han with EH were higher than in the normotensive population, and some EH patients showed matrilineal inheritance (Li et al., 2009; Liu et al., 2009).

Adiponectin (APN) secreted by adipose tissue has obvious cardioprotective effects, and hypoadiponectinemia is a risk factor of various cardiovascular diseases. In mice overexpressing the *APN* gene, APN reduced blood pressure by improving endothelial dysfunction (Zhu et al., 2008). Ruan et al. observed perivascular inflammation and vascular injury regulated by complement-mediated inhibition of APN in patients with hypertension (Ruan et al., 2017). Increasingly, studies are reporting a significant correlation between APN and mitochondrial function. Mitochondrial dysfunction can directly reduce the secretion of APN and may indirectly reduce APN synthesis via the endoplasmic reticulum stress response although supplementation of exogenous APN can improve mitochondrial function (Wang et al., 2013). APN can also promote mitochondrial production via the AdipoR1, AMPK/SIRT1, and PGC-1α pathways. In other words, there are negative correlations between APN and the occurrence of EH and between APN and mitochondrial dysfunction; the latter plays an important role in the occurrence and development of maternally inherited EH. Therefore, we speculate that the APN pathway is a potential mechanism of maternally inherited EH.

Matrilineal EH is related to mtDNA mutations, but the mechanism remains unknown. To solve this problem, we study the relationship between mtDNA mutations and EH and reveal the role and possible mechanism of APN in maternally inherited EH.

## Methods

### Subject, Inclusion, and Exclusion Criteria

The subjects were Chinese Han seen by the Department of Cardiology of the Chinese People's Liberation Army (PLA) General Hospital from July 1, 2013, to December 31, 2013. The subjects were not related by blood. The criteria for judging EH and maternally inherited EH pedigrees were based on the criteria in our previous study (Liu et al., 2014). In the non-maternally inherited EH pedigrees, the subject was an EH patient, and no matrilineal members within three generations were clearly diagnosed with EH. The admission criteria for the control group were no EH in any family member within three generations, including the subject.

Patients were excluded if they had secondary hypertension, severe heart failure, cardiomyopathy, rheumatic heart disease, congenital heart disease, another diagnosed mitochondrial disease, malignant tumors, hematological disease, hepatitis, human immunodeficiency virus, syphilis, or other infectious disease; were taking contraceptives or were pregnant or preparing for pregnancy within 6 months; or were judged as unsuitable to participate in the study by a clinician.

The study was approved by the Ethics Committee of Chinese PLA General Hospital, and all subjects provided informed consent. The 115 members who met the study conditions were divided into the maternally inherited EH (group A, *n* = 17), non-maternally inherited EH (group B, *n* = 65), and control (group C, *n* = 33) groups. All subjects completed a questionnaire about themselves and their family members.

### Whole Mitochondrial Gene Sequencing

From each subject, 400 μL whole blood was treated according to the SimGEN Blood DNA MiniKit instructions. DNA was extracted to measure optical density (OD) and was subjected to electrophoretic detection. Because the Cambridge Human Mitochondrial Gene has 16,569 bases, we divided the mitochondrial gene into 17 fragments and sequenced the entire mitochondrial gene. Then PCR amplification, purification, and extension were carried out, and the products were sequenced.

### Extraction and Culture of Human PBMCs and Plotting the Cell Growth Curve

Human peripheral blood mononuclear cells (PBMCs) were extracted and cultured in 10% FBS (Gibco^TM^), 1% penicillin/streptomycin (10,000 U/mL, Gibco^TM^), and 89% RPMI 1640 medium (Gibco^TM^). The state of cell growth was observed and photographed every day. The cells were counted regularly, and a cell growth curve was plotted.

### Determination of Plasma APN

Human plasma APN was quantified using an enzyme-linked immunosorbent assay (ELISA). The human ELISA kit was produced by Abcam. A standard sample or plasma, horseradish peroxidase-labeled antihuman APN monoclonal antibody, chromogenic agent, and terminator were added to solid-phase 96-well plates in turn. The degree of chromogenic reaction was positively correlated with the APN content of the plasma or standard sample. Using the absorbance corresponding to the concentration of a standard APN sample, a standard curve was drawn using the ELISA Calc regression/fitting calculation program provided by the reagent manufacturer and a four-parameter logistic curve fitting equation. The APN content in the sample was calculated from the absorbance.

### ATP, Caspase3/7, and ROS of Cells

The ATP and Caspase3/7 produced by cells were detected following the instructions of the CellTiter-Glo® Luminescent Cell Viability and Caspase-Glo® 3/7 Assays (Promega). The cell viability and apoptosis curves of each group were generated using the total number of cells as the abscissa and ATPase and Caspase3/7 as the ordinates. The fluorescent probe DCFH-DA was diluted in serum-free RPMI1640 and added to the cell suspension at a final concentration of 1 nM. The fluorescence was measured via flow cytometry; the total fluorescence equaled the amount of ROS produced. A serum-free RPMI1640 cell suspension that contained the same number of cells with no fluorescent probe was used as a blank control, and a positive control with the same number of cells was added with 50 μg/mL ROSup 10 min after adding the probe.

### RT-qPCR Analyses

Total RNA was extracted from myocardial tissues of mice using RNAprep Pure reagent and reverse transcribed to cDNA with the TIANScript cDNA First-Strand synthesis kit. Specific PCR primers for AdipoR1, AdipoR2, PGC-1α, and ERRα were synthesized by Tiangen Biotechnology. Then, q-PCR was conducted in 20 μL reaction volumes using a 7900HT Fast Real-Time PCR System (ABI, USA). The data were analyzed with Stratagene Mx3000 software. Agarose gel electrophoresis and the 2^−ΔΔ*CT*^ relative quantitative calculation formula were used to obtain the difference in mRNA transcription levels of the target gene.

### Statistical Methods

Spss21.0, Origin8.5, and GraphPad Prism8 were used for analyses and processing. Results are expressed as the mean ± SD. The data with normal distributions were compared between two groups using *t*-tests and among multiple groups using ANOVA. Otherwise, the rank sum test was adopted. *P* < 0.05 was defined as statistically significant, and *P* < 0.01 indicated that the difference was highly significant.

## Results

### Comparison of Mitochondrial tRNA Mutation Rate and Blood Biochemistry Between Maternally and Non-maternally Inherited EH Patients

**Figure 1:**
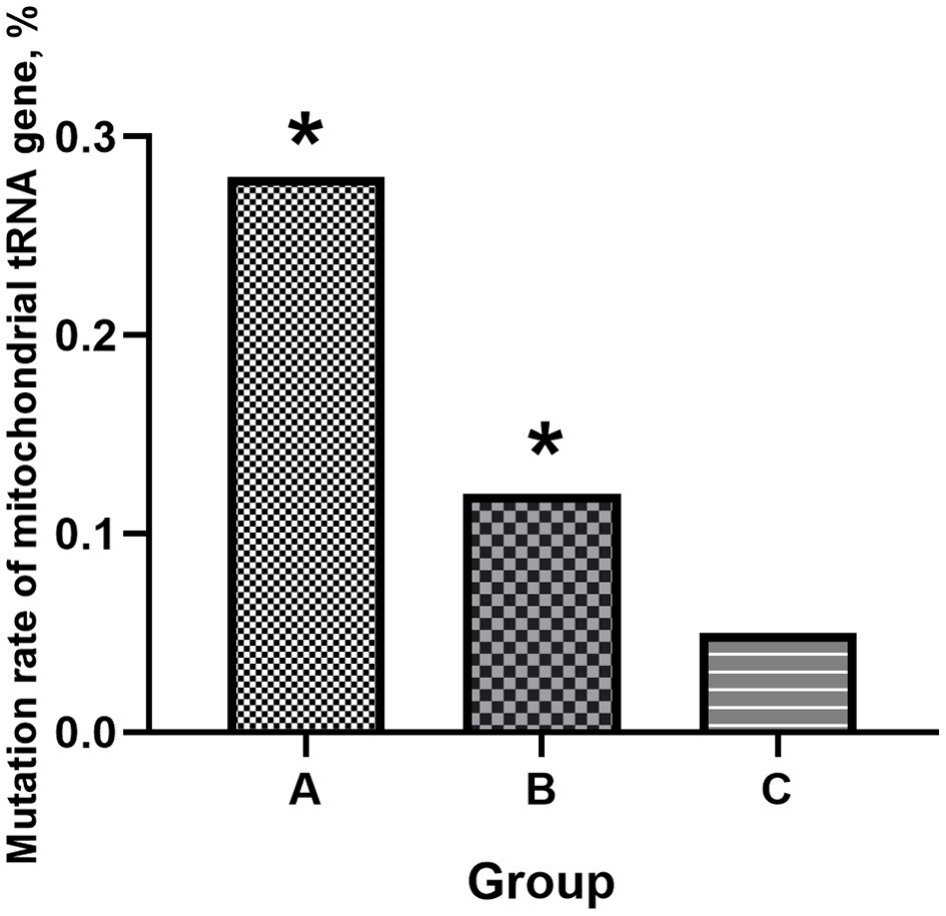
Mitochondrial tRNA gene mutation rates in the three groups. Compared to the controls, the two EH groups had higher mitochondrial tRNA mutation rates (*P < 0.05 vs. group C). Group A: maternally inherited EH, Group B: non-maternally inherited EH, Group C: control.

The detailed mitochondrial tRNA mutation is shown in Table 1. As the genes encoding mitochondrial tRNA contain 1504 bases, the total base number in each group is “*n* × 1,504.” Among them, the total mutation rates of the mitochondrial tRNA gene in groups A, B, and C were 0.28%, 0.12, and 0.05%, respectively (Figure 1). The mutation rate of the mitochondrial tRNA gene was significantly (*P* < 0.05) higher in groups A and B than in group C, and there were no significant differences between the two EH groups.

**Table 1:** Mitochondrial tRNA mutations within each group.

**Encoded product**	**Group A (*n* = 17)**	**Group B (*n* = 65)**	**Group C (*n* = 33)**
tRNA-Phe	5	11	1
tRNA-Val	0	0	0
tRNA-Leu (UUR)	0	0	1
tRNA-Ile	1	3	0
tRNA-Gln	2	2	1
tRNA-Met	3	3	0
tRNA-Trp	4	9	0
tRNA-Ala	8	36	8
tRNA-Asn	7	4	0
tRNA-Cys	12	6	2
tRNA-Tyr	6	2	0
tRNA-Asp	0	0	0
tRNA-Ser (UCN)	2	1	0
tRNA-Lys	1	3	1
tRNA-Gly	0	6	1
tRNA-Arg	0	2	0
tRNA-His	2	6	4
tRNA-Ser (AGY)	0	2	2
tRNA-Leu (CUN)	0	5	1
tRNA-Glu	6	11	1
tRNA-Thr	12	5	1
tRNA-Pro	3	9	0
Total mutations (S1)	74	126	24
Total base number (S2) (S2 = *n* × 1,504)	25,566	97,760	49,632
Total variation rate (S1/S2 × 100%)	0.28	0.12	0.048

### Mitochondrial tRNA 15910 C>T Mutation

**Figure 2:**
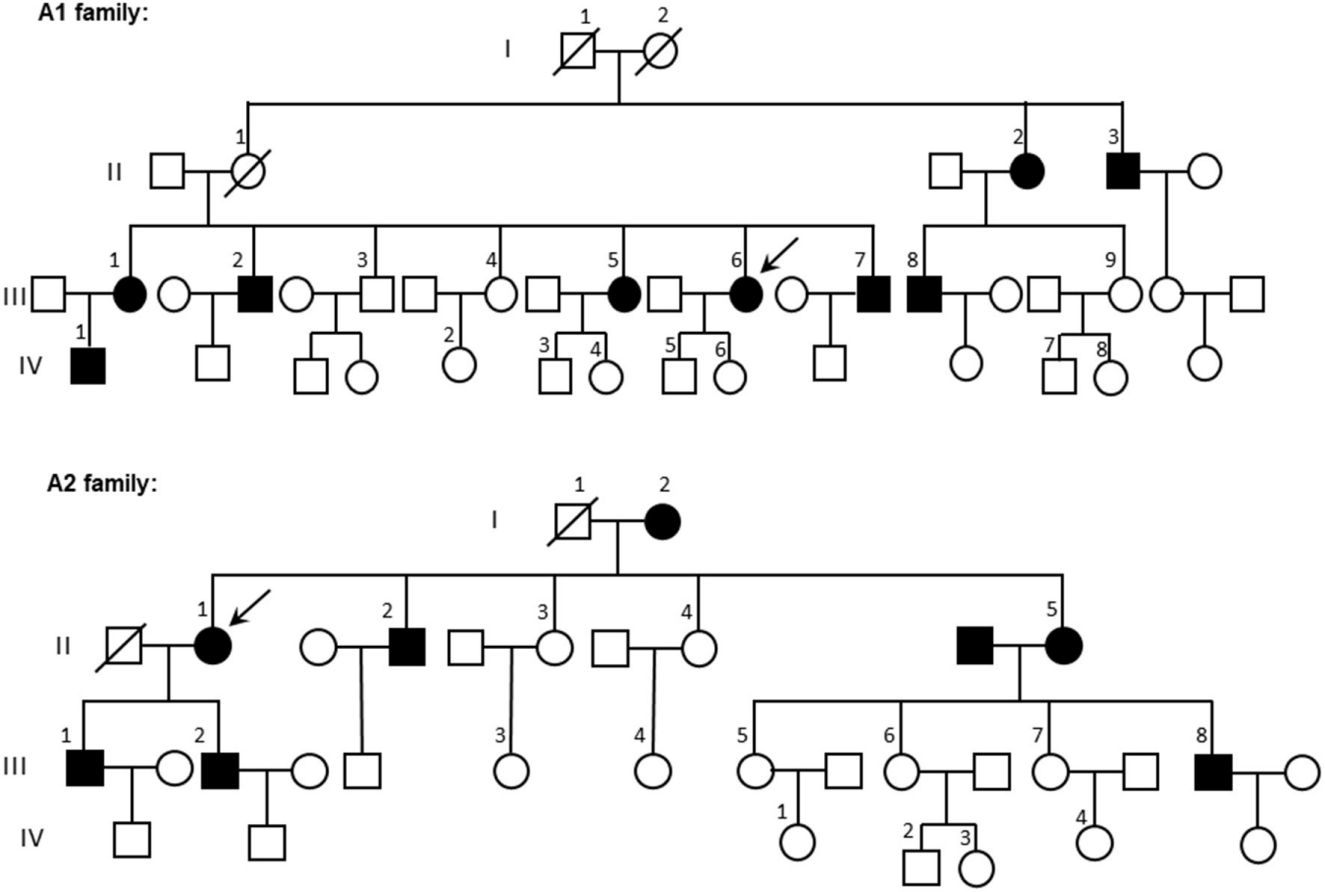
Family trees of pedigrees A1 and A2. Patients with hypertension are in black; the arrow indicates the proband. ◦, female; □, male.

The whole mitochondrial gene sequencing analyses of the 17 maternally inherited EH pedigrees (group A families) found that pedigrees A1 (Inner Mongolia, China) and A2 (Hebei, China) (Figure 2) had the same tRNA 15910 C>T mutation.

**Figure 3:**
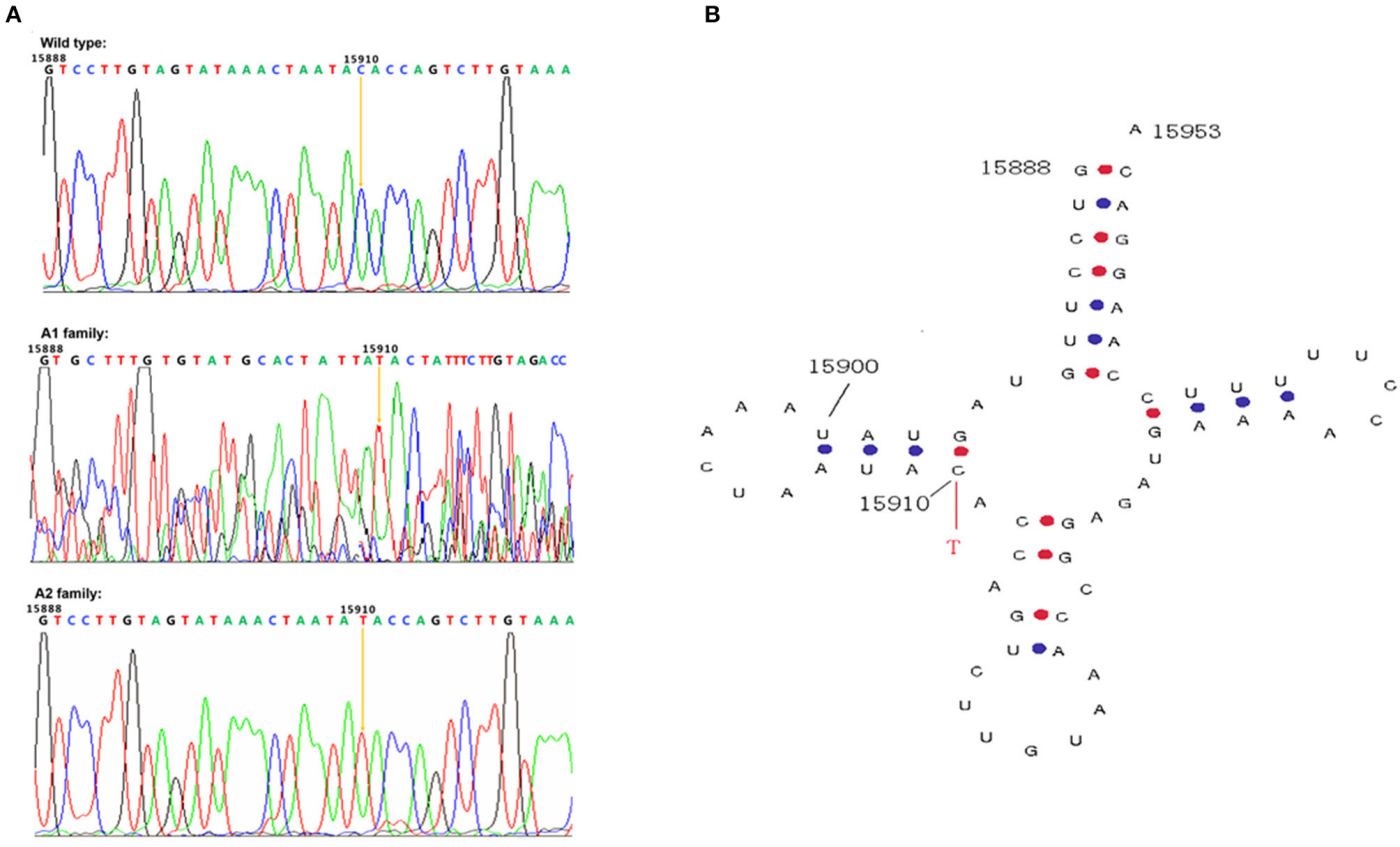
Mitochondrial gene sequencing and secondary structure of tRNA^Thr^ 15910. **(A)** The mitochondrial tRNA^Thr^ C>T mutation in families A1 and A2; **(B)** The first base pair in the D-loop formed by the C at 15910 and G at 15897 is disrupted by the tRNA^Thr^ C>T mutation.

Mitochondrial tRNA 15910 is located in the mitochondrial tRNA^Thr^ gene. The wild-type base at 15910 should be C but had mutated to T in the maternal members of pedigrees A1 and A2 (Figure 3A). Site 15910, which is a base in the D-loop arm of the mitochondrial tRNA^Thr^ gene (Figure 3B), forms the first base pair (C–G) in the D-loop with the G at site 15897. After mutation from C to T, the base pair cannot form, which may affect the function and stability of mitochondrial tRNA^Thr^.

### Characteristics of Pedigrees Carrying tRNA 15910 C>T

The occurrence of EH in pedigrees A1 and A2 was obviously matrilineal. Table 2 shows the incidence of EH in adult matrilineal members of families A1 and A2 with a total incidence of 59.3% and an incidence in males as high as 90.0%.

**Table 2:** Incidence of EH in adult matrilineal members of tRNA 15910 C>T family.

	**EH (incidence)**		**Average incidence (%)**
	**Male**	**Female**	
A1 family (*n =* 12,6 males)	5 (83.3%)	4 (66.7%)	75
A2 family (*n =* 15,4 males)	4 (100%)	3 (27.3%)	46.7
Average incidence	90.0%	41.2%	59.3

There were 16 adult matrilineal members with EH in two families. Based on sex and age, 40 eligible people were selected from the non-maternally inherited EH group (group B) and compared with the two pedigrees with tRNA 15910 C>T mutations (Table 3). The onset age of EH was significantly (*P* < 0.01) younger in matrilineal members of the two families carrying tRNA 15910 C>T (43.8 ± 8.6 vs. 52.3 ± 7.7 years for non-maternally inherited EH patients).

**Table 3:** Onset age of EH and blood pressure of tRNA 15910 C>T patients with EH and patients with non-maternally inherited EH.

	***n***	**Age**	**Age of onset**	**Maximum systolic blood pressure**	**Maximum diastolic pressure**
tRNA 15910 C>T	16	50.8 ± 14.9	43.8 ± 8.6	160.8 ± 20.3	99.2 ± 8.0
Non-maternally inherited EH	40	52.3 ± 6.9	52.3 ± 7.7	155.7 ± 17.3	95.3 ± 10.9
*P-*value		0.069	0.0007	0.38	0.15

### APN Level and APN-Related Molecules in Families Carrying tRNA 15910 C>T

To compare plasma APN levels, 24 subjects were divided into four groups of six each according to their family background and whether they had EH: (1) EH+Mu group (matrilineal members of family A2 carrying tRNA 15910 C>T and suffering from EH); (2) Mu group (matrilineal members of family A2 with tRNA 15910 C>T, but without EH); (3) EH group (from group B); (4) Control group (from group C). The ages of the Mu and control groups matched that of the other two groups.

**Figure 4:**
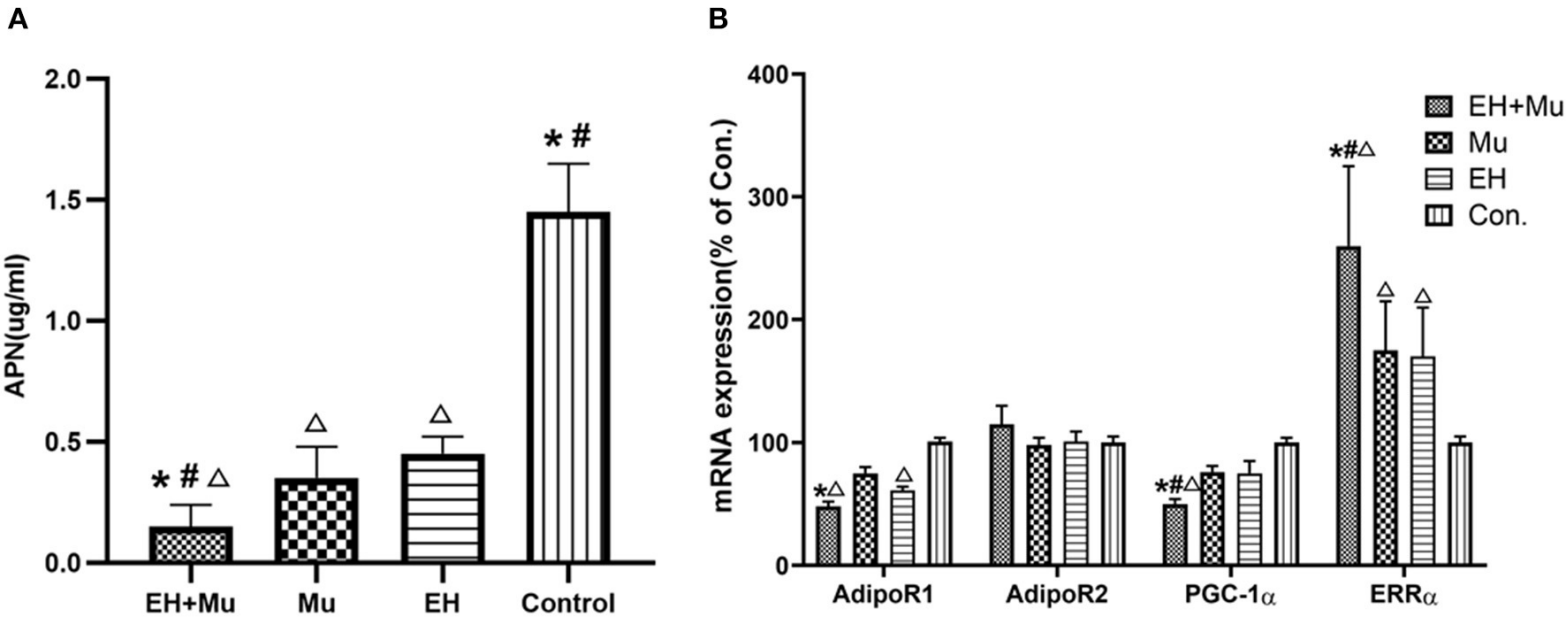
Plasma APN and expression of APN-related molecules. **(A)** The plasma APN levels of patients with or without tRNA 15910 C>T and/or EH (*P < 0.05 vs. Mu group; ^#^*P* < 0.05 vs. EH group; ^**Δ**^*P* < 0.05 vs. control group); **(B)** The mRNA levels of APN-related molecules in each group. (*P < 0.05 vs. Mu group; ^#^*P* < 0.05 vs. EH group; ^**Δ**^*P* < 0.05 vs. control group).

The average plasma APN level was measured in each group (Figure 4A) and was significantly lower in the EH+Mu group than in the other groups. The APN level in the Mu group was also significantly lower than that in the control. There were no significant differences between the Mu and EH groups.

PBMCs were extracted and cultured to analyze the AdipoR1/2, PGC-1α, and ERRα mRNA and protein expression in each group (Figure 4B). The expression of AdipoR1 mRNA was 52.5% lower in the EH+Mu group than in the controls and was 36.0% lower than that in the Mu group. The *PGC-1*α gene expression in each group was consistent with the change in direction of AdipoR1. There were no significant differences in AdipoR2 mRNA among groups. The expression of the *ERR*α gene in the EH+Mu group was significantly higher than in the other three groups. The expression of the *ERR*α gene in the Mu and EH groups was also significantly higher than in the controls.

### PBMC Growth and Apoptosis in tRNA 15910 C>T Carriers

**Figure 5:**
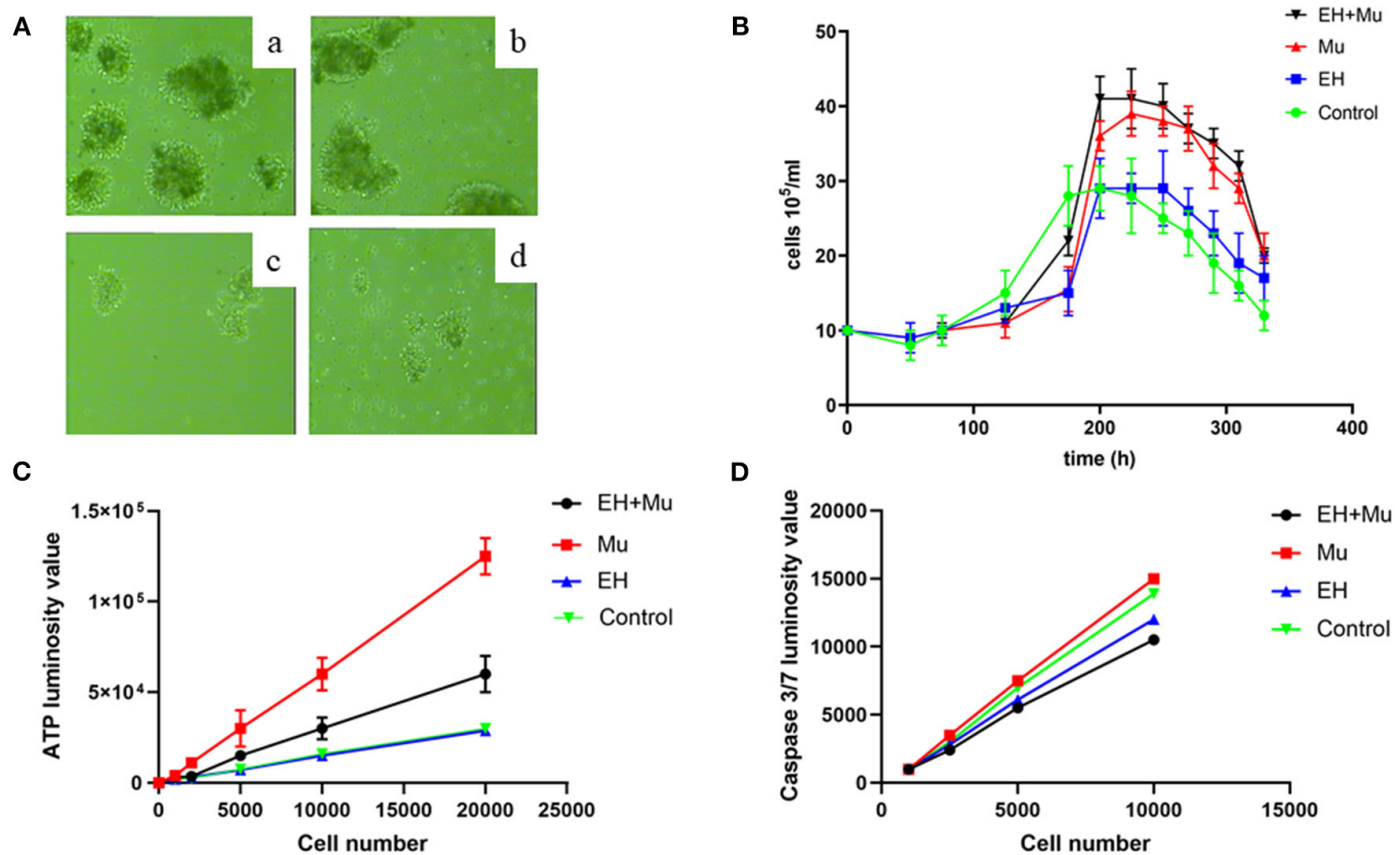
PBMCs growth and apoptosis in each group. **(A)** PBMC growth observed at 100× magnification on day 7; (a) EH+Mu; (b) Mu; (c) EH; (d) Control; **(B)** The PBMC growth curves in each group; **(C)** The generation of ATP in each group; **(D)** Caspase 3/7 apoptosis in each group.

The PBMCs of the EH+Mu and Mu groups proliferated *in vitro* (Figure 5A). The PBMC growth curve (Figure 5B) indicated that the maximum cell proliferation in the EH+Mu and Mu groups was about four times that in the EH and control groups. The PBMC viability curve of each group showed that PBMC activity was strongest in the Mu group with a significant difference in ATP production compared to the other three groups. Cell viability was significantly higher in the EH+Mu group than in the EH and control groups (Figure 5C). However, there were no significant differences in the Caspase3/7 apoptosis curve of PBMCs among the different groups (Figure 5D).

### Changes in the ROS of PBMCs Carrying tRNA 15910 C>T

**Figure 6:**
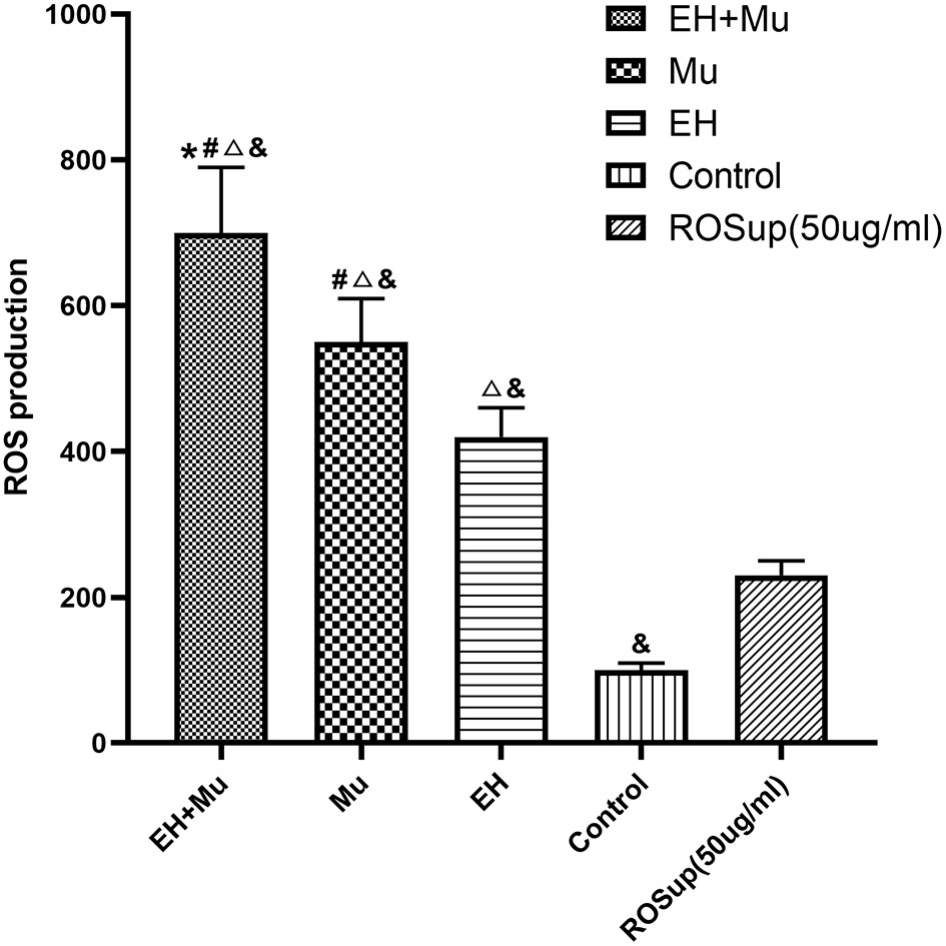
Comparison of ROS produced by cells in each group. The elevated ROS production in groups carrying tRNA 15910 C>T mutation (*P < 0.05 vs. Mu group; ^#^*P* < 0.05 vs. EH group; ^**Δ**^*P* < 0.05 vs. control group; ^**&**^*P* < 0.05 vs. ROSup [50 μg/mL]).

Comparing groups, the EH+Mu group produced significantly more ROS than the other three groups (Figure 6). The Mu group produced significantly more ROS than the EH, positive control (ROSup 50 μg/mL), and control groups. ROS generation in the EH group was also significantly higher than that in the control and positive control groups.

## Discussion

Two pedigrees with maternally inherited EH carried the same mitochondrial tRNA 15910 C>T mutants. Analyses of all matrilineal members of these two families show that the incidence of hypertension is as high as 59.3% although the latest survey reports that the prevalence of hypertension in Chinese adults is 23.2% and that of adult males is 24.5% (Wang et al., 2018). The incidence of hypertension in all adult matrilineal members of the two families is significantly (*P* < 0.05) higher than that in the population, suggesting that the mitochondrial tRNA 15910 C>T mutation is closely related to the occurrence of maternally inherited hypertension. Moreover, the onset age in the matrilineal members is younger than that of non-maternally inherited EH, and it decreased by generation, which might be related to the heterogeneity and homogeneity of mtDNA and the genetic mode of maternally inherited EH. In theory, the effects of matrilineal inheritance on EH should increase over the generations because EH can, in turn, induce mitochondrial mutations, which are transmitted to maternal offspring via maternal inheritance. Due to the genetic process of mtDNA, a heterogeneous mtDNA mutant may drift into a simple mtDNA mutant after one or two generations of matrilineal inheritance, and the mutation threshold increases, which makes mitochondrial functional damage more likely. Our results concur with the literature on EH (Zhu et al., 2016). Our analyses of mitochondrial tRNA 15910 C>T are horizontal analyses of biological evolution. Then, we analyzed the biological conservatism of this locus longitudinally. In the mitochondrial gene sequences of other mammals, the base of the corresponding human mitochondrial tRNA 15910 locus is C (Calvo et al., 2016), indicating that this locus is highly conserved, and the base at this locus plays an important role in maintaining the function and stability of mitochondrial tRNA^Thr^. The matrilineal members of two different families carrying mtDNAC15910T had a very high prevalence of EH, which shows maternal inheritance, suggesting that mitochondrial tRNA 15910 C>T is closely related to the occurrence of EH.

Furthermore, the ROS of matrilineal members carrying mitochondrial tRNA 15910 C>T was markedly increased, which was more obvious in cells from EH patients. Their AdipoR1 expression was significantly downregulated, and the plasma APN level was also significantly decreased, suggesting that the increase in ROS was negatively correlated with the expression of AdipoR1 and plasma APN. In addition, the APN level was positively correlated with the expression of peroxisome proliferator-activated receptor γ coactivator-1α (PGC-1α) and negatively correlated with the level of estrogen-related receptor alpha (ERRα).

People with other diseases caused by mitochondrial mutations had low APN levels (Ninomiya et al., 2016), and APN protected against incident hypertension independent of the body fat distribution (Peri-Okonny et al., 2017). Moreover, artificially induced mitochondrial dysfunction and excessive ROS production reduced APN secretion in adipose tissue (Wang et al., 2013). In APN-deficient mice, endothelium-dependent vasodilation was impaired, endothelial nitric oxide (eNOS) activity decreased, NO decreased, and systolic blood pressure was higher, which could be reversed by APN intervention (Zhu et al., 2008). APN has a cardioprotective effect, which is mainly mediated by adiponectin receptors (AdipoR1 and AdipoR2), and angiotensin II (Ang II) inhibits AdipoR1 expression in cardiomyocytes *in vivo* and *in vitro*, and the combination of Ang II (AT1 and AT2) and adiponectin receptors instigates the inhibition of the cytoprotective action of adiponectin receptors (Zha et al., 2017). Reducing the production of ROS can reverse the effects of Ang II on the expression of AdipoR1, indicating that ROS play a pivotal role in regulating Ang II in AdipoR1 expression. When mitochondrial function is insufficient, ROS production increases, which impairs APN secretion by adipocytes and downregulates AdipoR1 expression.

As a transcriptional activator of the nuclear receptor family, PGC-1α is the transcriptional activator of PPAR-γ, both of which regulate the expression of genes related to energy metabolism (Di et al., 2018). PGC-1α, which mainly regulates mitochondrial production and the homeostasis of mitochondrial copy number, is the main regulator of mitochondrial production downstream in the AMPK pathway (Dong et al., 2020). It regulates the dynamic balance between mitochondrial function and the energy produced, activates the antioxidant enzyme system, and scavenges ROS (Suntar et al., 2020). When PGC-1α is deleted, the activities of mitochondrial antioxidant enzymes decrease significantly, and ROS production and oxidative stress are both enhanced (Lu et al., 2010). APN and its receptor AdipoR1 can increase the expression of PGC-1α, enhance its activity, and enhance mitochondrial biosynthesis, which is consistent with our results (Pal et al., 2020).

ERRα, an orphan nuclear receptor, is an important regulator of blood pressure, mainly by affecting the balance of sodium and potassium in the kidneys (Wang et al., 2016). Loss of ERRα expression, whether stimulated by high sodium, low potassium, or high renin, leads to hypotension instead of hypertension. Most PGC-1α effector genes have ERRα binding sites, and PGC-1α not only cooperates with ERRα to activate the target gene transcription, but also regulates the ERRα activity (Brown et al., 2018). Therefore, tissues with high ERRα expression generally also overexpress ERRα. However, our results suggest that the expression of PGC-1α differs from that of ERRα. Comparing the relationship between the expression of PGC-1α and ERRα in the four groups, this phenomenon is seen in every group, and the proportions are similar, which should exclude the deviation caused by test error. Previous studies report that ERRα could regulate cardiovascular function without relying on PGC-1α. When congestive heart failure occurred, ERRα significantly decreased, and AMPKa2 activation increased the ERRα expression to improve cardiomyocyte mitochondrial function and heart failure, but the expression of PGC-1α did not increase (Hu et al., 2011). Histone deacetylation and PGC-1α expression downregulation also occurred in cardiomyocytes during the initial stage of hypoxia (within 12 h), and ERRα overexpression increased the PGC-1α expression (Ramjiawan et al., 2013). This indicates that ERRα can also regulate the expression of PGC-1α, and there may be a feedback mechanism between them. Hypoadiponectinemia downregulated PGC-1α expression and induced an increase in ERRα expression, which might be related to their mutual regulation.

In summary, we speculate that mitochondrial tRNA 15910 C>T impairs mitochondrial function, increases ROS production, downregulates the expression of APN and its receptor AdipoR1, and then suppresses the expression of PGC-1α and elevated ERRα via the mutual regulation between ERRα and PGC-1α, consequently resulting in further mitochondrial dysfunction and high blood pressure.

Cell proliferation is the main form of cell activity. At present, there are two methods to detect cell proliferation. One is a direct method to evaluate the proliferation ability of cells by directly measuring the number of cells undergoing division, such as the BrdU test. The other is an indirect method, namely the cell viability test, which evaluates cell proliferation by detecting the number of healthy cells in the sample, such as MTT and ATP detection. ATP is the direct energy source of cells, so the proliferation state of cells can be directly reflected by detecting the content of ATP in cells (Adan et al., 2016). The mitochondrial tRNA 15910 C>T mutation resulted in a significant increase in the cell proliferation rate, cell activity, ATP production, and ROS production as a byproduct in cultured PBMCs from each group, particularly in those with the mitochondrial mutation combined with EH, consistent with our previous research (Liu et al., 2017). However, there were no significant differences in the Caspase3/7 apoptosis curve among groups, demonstrating that mitochondrial tRNA 15910 C>T mainly affects proliferation and not apoptosis.

The analyses of the mtDNA mutation and EH show that the mutation rates of the mitochondrial tRNA gene in the control, non-maternally inherited EH, and maternally inherited EH groups were 0.048, 0.12, and 0.28%, respectively. Compared to the controls, there were significantly more mitochondrial tRNA gene mutations in the EH groups (*P* < 0.05), which concurs with the literature (Zhu et al., 2018), suggesting that there is a close correlation between the occurrence of EH and the mitochondrial tRNA gene mutation. The mechanism might be related to the increase in Ang II in hypertensive people. Studies show that Ang II can increase intracellular ROS, and the interaction between them affects mtDNA damage (Wilcox et al., 2019; Zhang et al., 2019). However, a difference in the mitochondrial tRNA mutation rate between the maternally inherited and non-maternally inherited EH populations could not be elucidated, which might be due to the small sample size, particularly subjects with maternally inherited hypertension. The effect of matrilineal inheritance on EH was 20.73%, lower than that in the Framingham Heart study (35.2%) (Yang et al., 2007). Our study definitely had a small sample, and the Framingham study enrolled 1,593 families.

As mitochondria play an important role in the pathogenesis of hypertension, the treatments targeting mitochondria to decrease blood pressure have been research hot spots. Coenzyme Q10 can prevent hypertension through antioxidant properties, preserving NO, and boosting the production of the prostaglandin prostacyclin (Yang et al., 2015). Eisenberg et al. discovered that oral spermidine facilitated cardioprotective effects in elderly mice through strengthening cardiac autophagy and mitophagy. In salt-sensitive rats, spermidine treatment also delayed the progression of hypertensive heart disease in company with decreased arterial blood pressure (Eisenberg et al., 2017).

There are some limitations to this study. First, the sample size is small. A sufficient sample size helps to ensure the accuracy of the research results, and we analyzed only 115 subjects and families. Second, this is a retrospective study, and it is necessary to alter the key genes in animal and cell experiments to verify their effects.

The maternal inheritance of mtDNA can help us predict the risk for diseases related to mtDNA mutations in offspring and lead to prevention, early diagnosis, and timely treatment. Although there is no radical cure for EH, early initiation of treatment and blood pressure control can reduce the complications of hypertension. We find a new mitochondrial tRNA mutation 15910 C>T related to maternally inherited EH, reveal part of the mechanism by which it led to the occurrence and development of maternally inherited EH, and discuss the role of APN in it and its possible mechanism. This provides a new theoretical and experimental basis for the study of maternally inherited EH and probably a new target for the prevention and treatment of it.

## Data Availability Statement

All datasets generated for this study are included in the article/supplementary material.

## Ethics Statement

The studies involving human participants were reviewed and approved by the Ethics Committee of Chinese PLA General Hospital, Chinese People's Liberation Army General Hospital. The patients/participants provided their written informed consent to participate in this study.

## Author Contributions

JB and QM completed the experiments. YLa, YC, SM, JL, CL, ZF, XL, YH, and YLi contributed to the conception, drafted the manuscript, critically revised the manuscript, gave final approval, and agreed to be accountable for all aspects of work ensuing integrity and accuracy. All authors contributed to the article and approved the submitted version.

## Conflict of Interest

YLa was employed by the company Hainan LANBO Health Management Co. Ltd. The remaining authors declare that the research was conducted in the absence of any commercial or financial relationships that could be construed as a potential conflict of interest.
